# A Wireless Passive LC Resonant Sensor Based on LTCC under High-Temperature/Pressure Environments

**DOI:** 10.3390/s150716729

**Published:** 2015-07-10

**Authors:** Li Qin, Dandan Shen, Tanyong Wei, Qiulin Tan, Tao Luo, Zhaoying Zhou, Jijun Xiong

**Affiliations:** 1 Key Laboratory of Instrumentation Science & Dynamic Measurement, Ministry of Education, North University of China, Tai Yuan 030051, China; E-Mails: qinli@nuc.edu.cn (L.Q.); sdd19900313@163.com (D.S.); 18334792310@163.com (T.W.); 18935157540@163.com (T.L.); 2 Science and Technology on Electronic Test & Measurement Laboratory, North University of China, Tai Yuan 030051, China; 3 State Key Laboratory of Transducer Technology, Department of Precision Instrument and Mechanology, Tsinghua University, Beijing 10084, China; E-Mail: zhouzy@tsinghua.edu.cn

**Keywords:** LC resonant sensor, DuPont 951 ceramic, dielectric constant, Young’s modulus, compensation structure

## Abstract

In this work, a wireless passive LC resonant sensor based on DuPont 951 ceramic is proposed and tested in a developed high-temperature/pressure complex environment. The test results show that the measured resonant frequency varies approximately linearly with the applied pressure; simultaneously, high temperature causes pressure signal drift and changes the response sensitivity. Through the theoretical analysis of the sensor structure model, it is found that the increase in the dielectric constant and the decrease in the Young’s modulus of DuPont 951 ceramic are the main causes that affect the pressure signal in high-temperature measurement. Through calculations, the Young’s modulus of DuPont 951 ceramic is found to decrease rapidly from 120 GPa to 65 GPa within 400 °C. Therefore, the LC resonant pressure sensor needs a temperature compensation structure to eliminate the impact of temperature on pressure measurement. Finally, a temperature compensation structure is proposed and fabricated, and the pressure response after temperature compensation illustrates that temperature drift is significantly reduced compared with that without the temperature compensation structure, which verifies the feasibility the proposed temperature compensation structure.

## 1. Introduction

Low-temperature co-fired ceramic (LTCC), developed by the Hughes company in 1982, is a new type of ceramic material. LTCC, which is mainly composed of ceramic particles, glasses, and organic composition, can form a green tape of uniform thickness and density. Through the standard LTCC fabrication process, including steps such as punching, screen printing, stacking, and co-firing, a high-density circuit structure can be fabricated [1,2,3]. Because LTCC exhibits excellent merits for miniaturization, high integration, and compatibility, this technique has been widely used in fields such as high-frequency and microwave signals, especially combined with wireless passive sensing technology, making it widely used in high-temperature pressure measurement [4,5].

Wireless passive pressure sensors based on LTCC were first developed at the Georgia Institute of Technology in 1999. By introducing an air cavity inside the ceramic, the sensor resonant frequency was made sensitive to external pressure [6]. In 2002, Fonseca improved this structure and made this sensor applicable for a higher temperature range [7]. In the following years, the Vienna Technology University (Austria) and Novi Sad University (Serbia) proposed a pressure sensor structure that completely embedded the inductor and capacitor into the ceramic body, in order to protect the sensor from high temperature and corrosive environments [8]. Since 2010, North University of China improved the sensor performance by introducing a scarified layer technology during lamination and by improving the structure without an air exhaust hole. Further, it has realized pressure measurements under 600 °C [9].

The principle and fabrication process of an LC resonant sensor based on DuPont 951 ceramic are first introduced in this paper. Then, the fabricated sensor is tested on the developed high-temperature/pressure complex test system. The results indicate that high temperature causes drift and changes in the response sensitivity of the measured signal. From the in-depth theoretical analysis of the sensor structure model, it is found that high temperature causes an increase in the ceramic dielectric constant, and a decrease in the ceramic Young’s modulus. These results indicate that temperature compensation is needed for the LC resonant sensor, which can eliminate the influence of temperature on pressure measurement.

## 2. Fabrication and Measurement Based on DuPont 951 Ceramic

### 2.1. Sensor Fabrication

**Figure 1:**
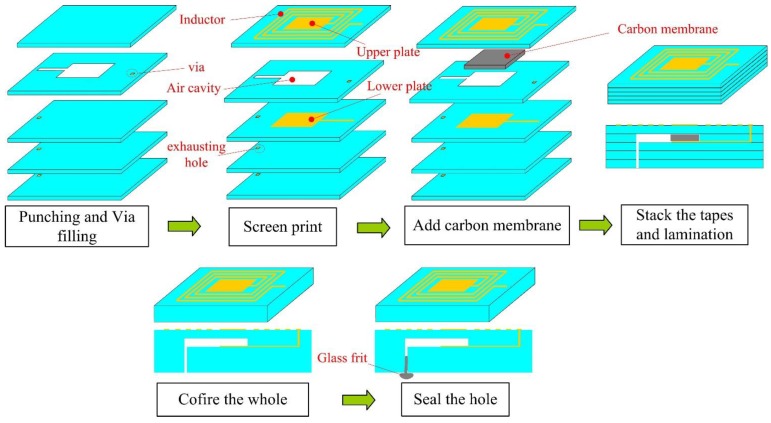
Main fabrication processes of the pressure sensor.

**Figure 2:**
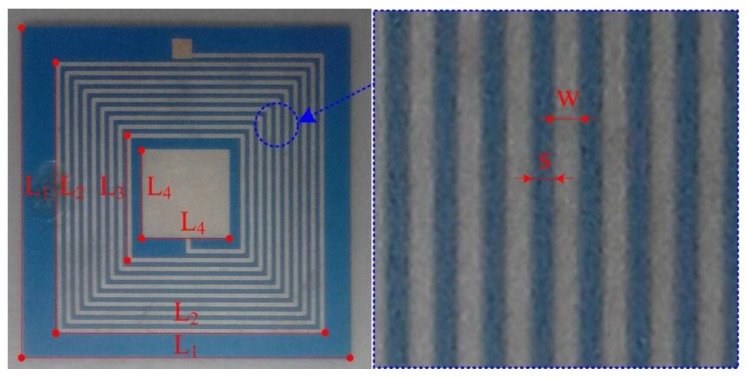
Fabricated pressure sensor.

Figure 1 shows the main fabrication processes of the pressure sensor, which is composed of five layers of green tape. First, the via, exhaust hole, and air cavity are formed by punching on corresponding layers, then the via is filled with metal silver, in order to connect the patterns on different layers; Second, layers 1 and 3 are screen printed with the corresponding sliver patterns; Third, a carbon membrane is added into the cavity, to protect the sensitive membrane from collapse. Fourth, all the tapes are stacked in proper order and laminated into a unit. Fifth, the whole structure is sintered in a furnace according to the time-temperature curve set in advance; during the sintering, the carbon membrane is evaporated into the air through the exhaust hole; Finally, the structure is co-fired in a furnace again, to seal the exhausting hole by glass frit. The fabricated pressure sensor is illustrated in Figure 2 and the main sensor dimensions are listed in Table 1.

**Table 1:** Main dimensions of the fabricated pressure sensor.

Variable	Parameter Name	Value (mm)
L_1_	Substrate length	33.8
L_2_	Inductor outer diameter	28.1
L_3_	Inductor inner diameter	13.5
L_4_	Capacitive plate length	9.0
W	Inductor wire width	2.56
S	Inductor wire spacing	2.25

### 2.2. Sensor Principle

**Figure 3:**
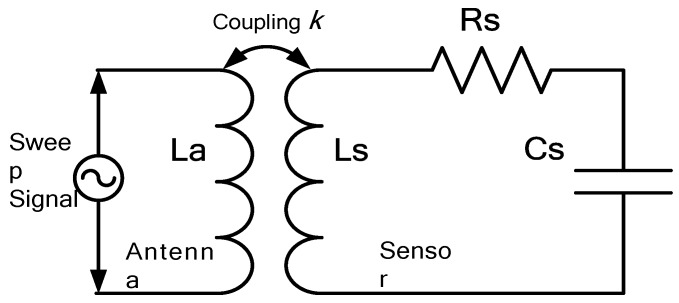
Equivalent measurement principle of the fabricated sensor.

The sensor measurement principle is shown in Figure 3. The fabricated sensor can be simplified as a resistor-inductor-capacitor (R-L-C) series connection, where the resistance is caused by the coil wire. The inductor coil is coupled to the antenna coil, which sends out an alternating current sweep frequency signal of a certain bandwidth. When the frequency of the sweep signal approximates the sensor self-resonant frequency *f*_0_, the impedance information of the antenna, including the real part, imaginary part, amplitude, and phase, will mutate. By extracting the antenna impedance information, the sensor frequency can be obtained. Generally, we focus on the impedance phase part of the antenna; the mutated peak frequency *f*_min_ has the following relation with *f*_0_ [10]:

(1) $$f_{\min}=f_{0}\left(1+\;\frac{k^{2}}{4}\;+\;\frac{1}{8Q^{2}}\right)$$

where *k* is the coupling coefficient between the antenna coil and the inductor coil, and *Q* denotes the sensor quality factor. Because the coupling between two coils is usually very weak, *k* is very small, while the quality factor *Q* is large. Thus, the difference between *f*_min_ and *f*_0_ can be neglected [11].

### 2.3. Sensor Measurement

**Figure 4:**
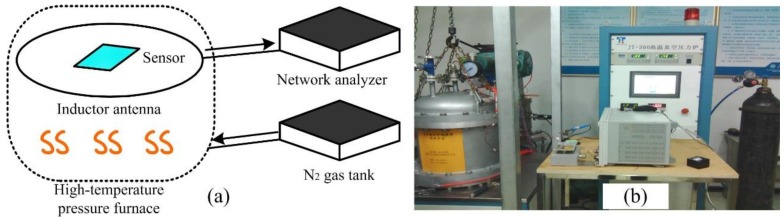
(**a**) Schematic and (**b**) physical diagrams of the high temperature/pressure complex measurement system.

In order to measure the fabricated sensor, a complex system for simultaneously high temperature and pressure measurement is developed. Figure 4 illustrates the schematic and the physical diagrams of the measurement system. The sensor is heated from room temperature to 400 °C at a 50 °C step; when the temperature is kept constant, the furnace absolute pressure is raised by nitrogen gas from 1.7 to 3 bar.

**Figure 5:**
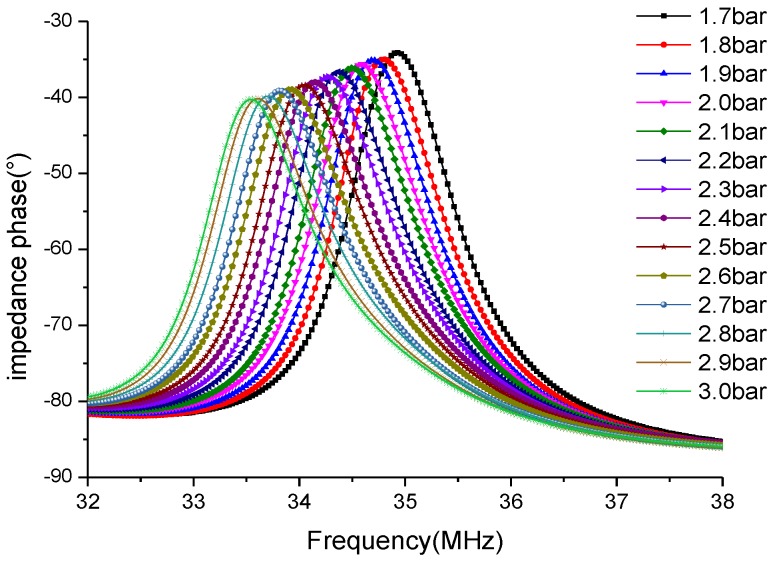
Readout signals of different gas pressures at 20 °C.

**Figure 6:**
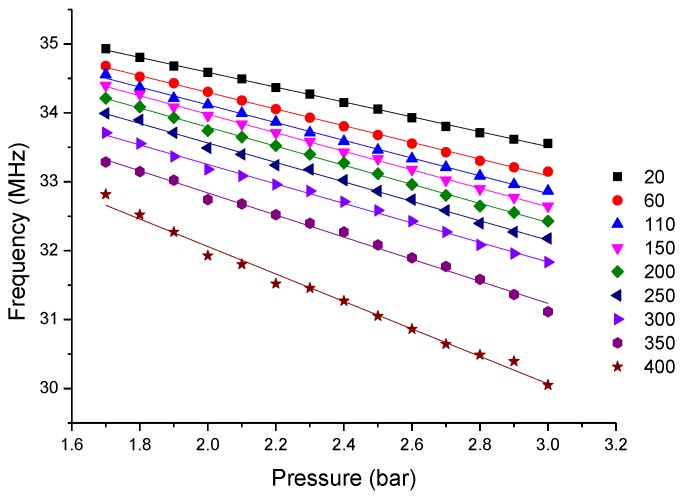
Extracted frequency versus pressure at different temperatures.

Figure 5 shows the readout signals of different gas pressures at 20 °C. It is found that a positive peak occurs in every measurement curve and the peak frequency shifts leftward monotonously. The extracted peak frequency versus different pressures under different temperatures is illustrated in Figure 6.

The measurement results, shown in Figure 6, illustrate that for the same temperature, the measured resonant frequency decreases approximately linearly with the increase of exerted pressure. Meanwhile, the temperature is found to have a significant influence on the pressure measurement. It is found that for the same pressure, the measured frequency drifts downward at elevated temperatures; simultaneously, the elevated temperature causes enhanced sensor pressure response sensitivity.

## 3. Analysis of Sensor Structure Model

The resonant frequency of the LC sensor is defined as:

(2) $$f_{0}=\frac{1}{2\pi \sqrt{L_{s}C_{s}}}$$

**Figure 7:**
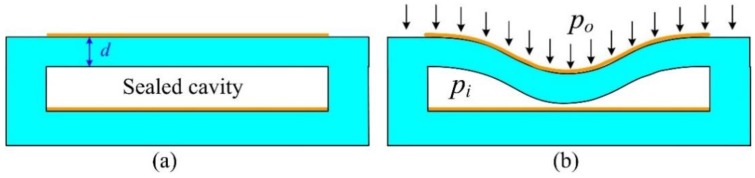
Pressure sensor model (**a**) before deformation (**b**) after deformation.

As shown in Figure 7a, there is a layer of ceramic and a layer of air cavity between two capacitor plates; thus, the sensor capacitance before deformation is calculated as:

(3) Cs0=ε0sd+dεr

where *d* is the thickness of the layer of ceramic tape, *ε_0_* denotes the permittivity of vacuum, and *ε_r_* represents the relative permittivity of the substrate ceramic.

When the exerted pressure deforms the sensitive film, shown in Figure 7b, the capacitance *C_s_* is given as [12]:

(4) $$C_{s}=\frac{C_{s0}{\text{tanh}}^{-1}\left(\sqrt{\beta }\right)}{\sqrt{\beta }}$$

where:

(5) β = 12bΔpa4(1−ν2)Ed4(1+1εr)

In Equation (5), *b* is a coefficient related to the shape of the capacitor plates, *a* denotes the length of the short side of capacitor plate, and *v* and *E* represent the Poisson ratio and Young’s modulus of the substrate ceramic, respectively. Δ*p* is the pressure difference between the exerted nitrogen pressure *p_o_* and internal pressure *p_i_* of the sealed cavity, and is calculated as:

(6) $$\Delta p=p_{o}-p_{i}$$

Combining Equations (5) and (6), the coefficient *β* is roughly calculated as 10^−1^ in order of magnitude. When Taylor expansion is performed on Equation (4) and the higher-order terms are omitted, *C_s_* is expanded as:

(7) $$C_{s}=\;\;C_{s0}\left(1\;+\;\frac{\beta }{3}\right)$$

Substituting Equation (7) into Equation (2), *f*_0_ is derived as:

(8) $$f_{0}=\;\frac{1}{2\pi \sqrt{L_{s}C_{s0}\left(1\;+\;\frac{\beta }{3}\right)}}$$

Because *β* is small, Equation (8) undergoes further Taylor expansion and the higher-order terms are omitted once again; simultaneously combining Equations (5) and (6), *f*_0_ is calculated as:

(9) $$f_{0}=A\left(D-Cp_{o}\right)$$

(10) $$D=B\;+\;Cp_{i}$$

where:

(11) A=12πdLsε0s, B=(1 +1εr), C=2ba4(1−v2)Ed4(1 +1εr)

When the temperature rises, the sensor inductance *L_s_* is seen as invariable according to previous experiments [13]. High temperature will expand the ceramic size; the thermal expansion coefficient of DuPont 951 ceramic is 5.8 × 10^−6^/°C. The ratio of plate gap *d* to plate area *s* is calculated to be reduced by 0.00058% within 400 °C. Therefore, the change of coefficient *A* in Equation (11) can be ignored; namely, it can be seen as a constant, within the measuring temperature range.

Figure 6 illustrates that for the same pressure, the measured frequency decreases at elevated temperatures, which indicates the decrease of coefficient *D* in Equation (9). When the temperature rises, the air within the sealed cavity expands to balance the exerted gas pressure. Thus, the air pressure *p_i_* inside the cavity approximately equals the external gas pressure *p_0_*. The coefficient *C*, the slope of the fitted curve, simultaneously increases at elevated temperatures. Therefore, it is inferred that the coefficient *B* in Equation (10) decreases at elevated temperatures. The expression of *B* illustrates that the relative permittivity of substrate ceramic *ε_r_* increases at elevated temperatures. By in-depth analysis, *ε_r_* is found to increase from 7.8 to 8.1 within 400 °C, which is why the measured frequency shifts downward at elevated temperatures for the same pressure.

When the temperature is invariable, the sealed air cavity is compressed at elevated nitrogen pressure, causing the air pressure *p_i_* of the sealed cavity to increase to some extent. It is shown in Figure 6 that the measured frequency has an approximately linear relation with exerted pressure *p_0_*; thus, it is reasonable to see the coefficient *C* as the slope of the fitted curve.

**Figure 8:**
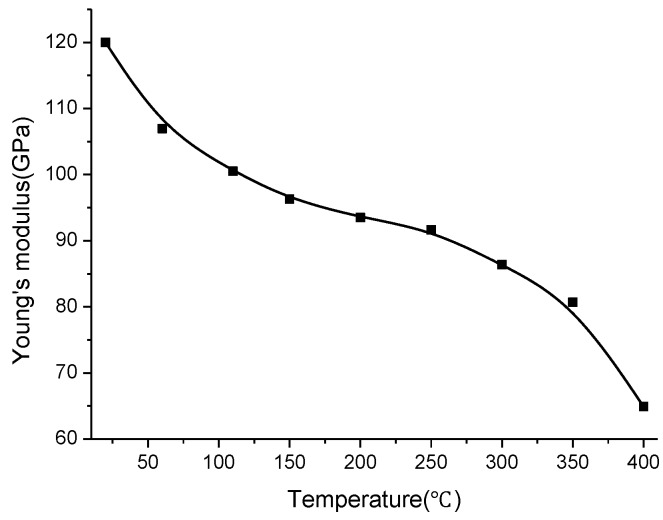
Calculated Young’s modulus of LTCC at different temperatures.

According to the expression of coefficient *C*, when the temperature increases from 20 °C to 400 °C, the ratio of *a^4^* to *d^4^* is seen as approximately invariable, whereas the variation of substrate ceramic permittivity causes the coefficient *C* to increase by a factor of only approximately 0.002, which differs from the substantial change of coefficient *C*. Therefore, it is inferred that the variation of substrate ceramic Young’s modulus could be the main reason causing the change. Ignoring the minimal change of dimensional proportion, variation of substrate permittivity etc minor factors, the coefficient *C* is found to be an inverse ratio with variable *E*. Thus, Young’s modulus of LTCC at different temperatures can be calculated approximately as:

(12) $$E=\frac{C_{0}}{C}E_{0}$$

where *E_0_* and *C_0_* are Young’s modulus of LTCC and slope of the measuring curve at room temperature respectively [14]. Young’s modulus of LTCC at different temperatures can be derived. Figure 8 illustrates that the Young’s modulus of LTCC always decreases at elevated temperatures, especially above 300 °C. The Young’s modulus of LTCC is calculated to decrease to 65 GPa within 400 °C, which illustrates that the material becomes softened at high temperatures, causing the sensitive membrane to deform further under the same pressure.

## 4. Temperature Compensation Structure of the Sensor

From the above analysis, it is known that temperature significantly affects the measurement of LTCC-based pressure sensors. Therefore, a temperature compensation structure is needed for the pressure sensor to eliminate the influence of temperature on pressure measurement. In particular, when exploring temperature compensation, focus should be on the effect of temperature on the variation of substrate ceramic permittivity and Young’s modulus.

**Figure 9:**
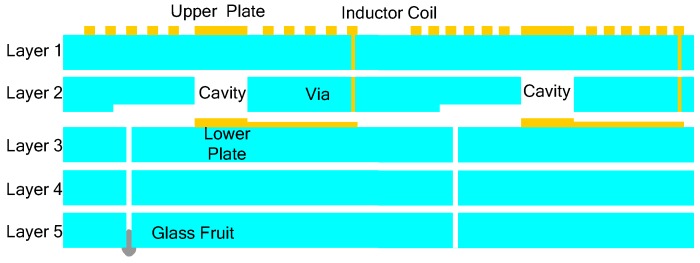
Temperature compensation structure for complex environments.

Figure 9 shows the proposed temperature compensation structure. The left LC loop is for pressure measurement, which is the same as the structure shown in Figure 1. Meanwhile, another LC loop, next to the left LC loop, is introduced for temperature measurement. The temperature capacitor structure is the same as that of the left sensor, which contains a layer of ceramic and a layer of air cavity; the two inductor shapes are different, to keep the two measured frequencies apart. The right via has no sealant, and the permittivity of LTCC is known to increase monotonously at elevated temperatures; thus, the right LC loop can be used for measuring temperature.

Because the two inductor coils do not overlap, the coupling between the sensor coils is negligible. The influence of crosstalk between the sensor coils can be ignored; thus, the resonant frequency of the temperature sensor is calculated as:

(13) f1=12πLs2ε0sd+dεr

Combining Equation (13) with Equations (4), (5) and (8), the exerted pressure can be derived as:

(14) $$p_{0}=\frac{\pi^{2}\varepsilon_{0}sd^{3}}{ba^{4}\left(1-\nu^{2}\right)}E_{\left(t\right)}\left(\frac{f_{1}^{4}L_{s2}^{2}}{f_{0}^{2}L_{s}}-f_{1}^{2}L_{s2}\right)\text{ + }p_{i}$$

By the right LC loop resonant frequency *f*_1_, the temperature can be obtained; thus, Young’s modulus *E*_(t)_ for the changed temperature is acquired. By substituting *E*_(t)_ and the two measured frequencies *f*_0_ and *f*_1_ into Equation (14), the external pressure after temperature compensation is obtained; thereby, the impact of high temperature on the sensor pressure measurement can be eliminated.

**Figure 10:**
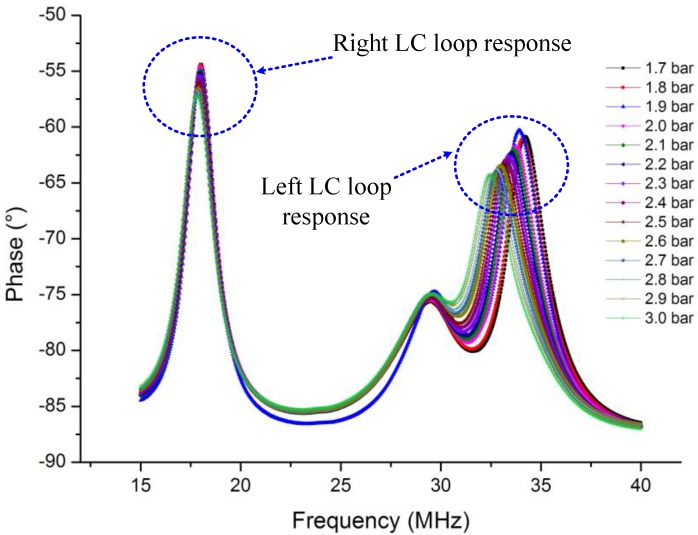
Proposed compensation structure pressure response in 200 °C.

In order to prove the above analysis, the proposed temperature compensation structure of Figure 9 is fabricated and tested under the same developed high-temperature/pressure complex environments. Figure 10 illustrates the pressure response curve of the compensation structure at 200 °C; the right peak is the response of the pressure sensing element, and the left peak is the response of the temperature sensing element.

**Figure 11:**
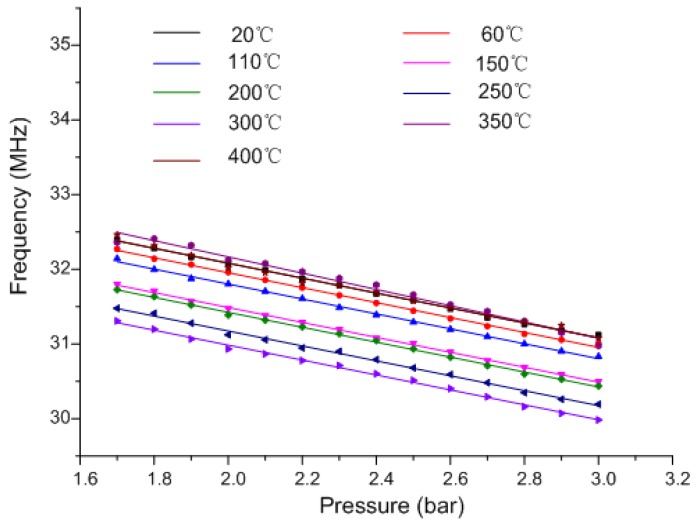
Measured curve of the pressure sensing element after temperature compensation.

The two extracted frequencies are substituted into Equation (14), and the pressure sensing element response after temperature compensation is shown in Figure 11. The influence of temperature on pressure measurement is found to decrease obviously compared with that in Figure 6. However, the temperature drift cannot be completely eliminated; the main reason is the negligence of coupling between the inductors and calculation errors of variables (such as inductances).

## 5. Conclusions

A wireless passive LC resonant sensor was fabricated based on DuPont 951 ceramic in this study, and it was tested in a developed high-temperature/pressure complex environment. It was found that the measured resonant frequency decreases approximately linearly with applied pressure, and at the same time the temperature causes pressure signal drift and changes in response sensitivity. To analyze the main reason that temperature affects the pressure signal, the theory was analyzed in detail. It was determined that under high temperature, the increase of DuPont 951 ceramic dielectric constant is the reason for the pressure signal drifts downward, and the decrease of Young’s modulus is the reason the enhanced response sensitivity. The DuPont 951 ceramic Young’s modulus was found to decrease rapidly to 65 GPa within 400 °C by calculation. Thus, a pressure sensor based on DuPont 951 ceramic needs temperature compensation when used in high-temperature environments. Finally, a compensation structure was proposed and fabricated; by tentative experiments, the feasibility of such a compensation structure was verified.
